# A meta-contrastive learning approach for clinical drug-drug interaction extraction from biomedical literature

**DOI:** 10.1371/journal.pcbi.1013722

**Published:** 2025-12-05

**Authors:** Yaxun Jia, Zhu Yuan, Lian Zhu, Zuo-lin Xiang

**Affiliations:** 1 Department of Radiation Oncology, Shanghai East Hospital, Tongji University School of Medicine, Shanghai, China; 2 Department of Information Management, The National Police University for Criminal Justice, Baoding, China; 3 Department of Radiation Oncology, Shanghai East Hospital Ji’an Hospital, Jian, China; Northwestern Polytechnical University, CHINA

## Abstract

Drug–drug interactions (DDIs) are a significant source of adverse drug events and pose critical challenges to patient safety and clinical decision-making. Extracting DDIs from biomedical literature plays an essential role in pharmacovigilance, yet remains difficult due to data sparsity and high annotation costs. This study presents BioMCL-DDI, a novel few-shot learning framework that integrates meta-learning with contrastive embedding strategies to enable efficient DDI extraction under limited supervision. BioMCL-DDI jointly optimizes prototype-based classification and supervised contrastive representation learning within a unified architecture. The model captures both intra-class compactness and inter-class separability, enhancing its generalization in sparse biomedical settings. We evaluate BioMCL-DDI on three benchmark datasets: DDI-2013, DrugBank, and the more recent TAC 2018 DDI Extraction corpus. The model achieves F1 scores of 87.80% on DDI-2013, 86.00% on DrugBank, and 74.85%/74.82% on the two official test sets of TAC 2018, consistently outperforming competitive baselines. Our model significantly outperforms state-of-the-art baselines in low-resource scenarios. BioMCL-DDI provides a scalable and effective solution for DDI extraction from biomedical texts, with strong potential for integration into clinical decision support systems and biomedical knowledge bases. All our code and data have been publicly released at: https://github.com/Hero-Legend/BioMCL-DDI.

## Author summary

Drug-drug interactions (DDIs) are a significant source of adverse drug events and pose critical challenges to patient safety. While existing drug databases offer valuable DDI information, they often struggle to keep pace with the rapidly expanding biomedical literature, leaving many new interactions unaddressed. To tackle this challenge, we developed BioMCL-DDI, a lightweight meta-contrastive learning framework designed for efficient DDI extraction from biomedical texts, especially in scenarios with sparse data. Our method integrates prototype-based classification with contrastive embedding strategies, jointly optimizing them within a unified architecture. This allows our model to generalize effectively even with limited annotated data. We evaluated BioMCL-DDI on two benchmark datasets, and our results show that our model significantly outperforms existing techniques in low-resource settings. BioMCL-DDI offers a scalable and effective solution for DDI extraction from biomedical texts, with strong potential for integration into clinical decision support systems and biomedical knowledge bases to enhance medication safety.

## Introduction

Drug–drug interactions (DDIs) are a critical factor in pharmacovigilance and clinical decision-making, as they can significantly alter the efficacy or safety of co-administered drugs. In clinical settings, especially among elderly patients or those undergoing polypharmacy, unrecognized DDIs can lead to adverse drug reactions (ADRs), increased hospitalization, and even mortality [1–3]. Thus, accurate and timely identification of potential DDIs is vital for enhancing medication safety and supporting personalized treatment regimens. While structured knowledge bases such as DrugBank offer manually curated DDI records, they are often incomplete or unable to keep pace with the rapid expansion of biomedical literature. This creates a pressing need for automatic DDI extraction systems that can identify emerging interactions from unstructured biomedical texts [4–9].

Recent advances in deep learning, particularly the introduction of transformer-based models such as BioBERT [11], have significantly improved the performance of biomedical relation extraction tasks, including DDI classification. However, these models typically require large-scale annotated corpora, which are rarely available in real-world scenarios—especially when dealing with new drug compounds or infrequent interaction types. Under such low-resource conditions, traditional supervised learning methods often struggle with data sparsity, class imbalance, and limited generalization to unseen relation types. Few-shot learning has emerged as a promising solution to address these challenges. Meta-learning techniques such as Prototypical Networks enable rapid adaptation to new classes using only a few labeled instances, while contrastive learning facilitates more discriminative embedding spaces through pairwise representation alignment. However, existing few-shot approaches often suffer from two practical limitations: (1) meta-learning typically relies on episodic task sampling, which increases training complexity and limits scalability; and (2) contrastive learning is often applied only during pretraining or as an auxiliary loss, thus underutilizing its potential in few-shot supervised settings.

To address these issues, we propose BioMCL-DDI, a unified meta-contrastive learning framework for few-shot DDI extraction under sparse supervision. Unlike previous methods that treat meta-learning and contrastive learning as separate objectives, our approach introduces a joint optimization strategy in which prototype-based classification and instance-level contrastive embedding are co-regularized. Moreover, we design a lightweight meta-inspired regularization component that improves intra-class cohesion and inter-class separability without requiring episodic sampling or task-level adaptation. This architecture enhances scalability and robustness in DDI scenarios characterized by noisy class boundaries and highly imbalanced distributions. The model’s essential output is a classification of drug pairs into one of five predefined categories: DDI-false, DDI-effect, DDI-mechanism, DDI-advise, or DDI-int. Experimental results on the benchmark DDI-2013 dataset demonstrate that BioMCL-DDI achieves a new state-of-the-art F1 score of 87.80%. In addition, cross-domain evaluation on the DDI-DrugBank corpus confirms the generalizability of our model, achieving 86.00% F1 with only 100 labeled samples per class. More importantly, evaluation on the recent TAC 2018 DDI Extraction corpus further validates the robustness of our framework, where BioMCL-DDI attains F1 scores of 74.85% and 74.82% on the two official test sets, surpassing all competitive baselines. These results highlight the strong cross-domain transferability of our approach and its potential applicability to real-world biomedical texts such as structured product labels and regulatory documents. Through extensive ablation and embedding analysis, we further demonstrate that our dual-objective architecture leads to improved semantic clustering and relational discrimination in few-shot biomedical NLP tasks.

Our main contributions are summarized as follows:

- We propose BioMCL-DDI, a meta-contrastive learning framework that unifies prototype-based classification and contrastive embedding learning into a single supervised architecture for few-shot DDI extraction.
- We introduce a meta-inspired regularization mechanism that eliminates the need for episodic training and enhances representation consistency in the presence of class imbalance and limited supervision.
- We conduct comprehensive experiments on DDI-2013 and DDI-DrugBank datasets, demonstrating that BioMCL-DDI outperforms recent strong baselines in both in-domain and cross-domain scenarios.
- We provide ablation and embedding space analyses to investigate how each module contributes to semantic representation quality and fine-grained relation modeling.

## Related work

Drug–drug interaction (DDI) extraction has long been an important task in biomedical NLP, with research evolving from traditional rule-based systems to advanced deep learning methods.

Early approaches utilized convolutional and recurrent architectures to encode sentence-level dependencies. For example, CNN-based models such as DCNN [12], CNN [13], SCNN [14], and MCCNN [15] exploited syntactic and positional cues to improve feature locality. Meanwhile, LSTM-based methods including ATT-LSTM [16], DLSTM [17], and Skeleton-LSTM [18] focused on long-range contextual modeling and attention-guided extraction.

To further capture complex biomedical semantics, hybrid and graph-based architectures were introduced. Joint-ABLSTM [19], HRNN [20], and PM-BLSTM [21] incorporated hierarchical or multi-path structures, while GCNN [22], GRU-GCN [23], and SM-GCN [24] applied graph neural networks for relation-aware inference.

The introduction of pretrained language models marked a major breakthrough in DDI extraction. Models such as R-BERT [25], MEAT-BioBERT [26], and CDBERT [28] leveraged contextualized biomedical embeddings to enhance semantic understanding. Recent variants like IMSE [29], DREAM [30], and EMSI-BERT [31] further integrated molecular structures and domain-specific features to push the performance frontier.

Beyond text-only representations, a growing body of work has focused on integrating structured biomedical knowledge. DDIKG [32], DKPL [33], and BERT-MLRE [35] introduce drug-related priors through prompts or external graphs. Simultaneously, attention-guided graph architectures such as BBL-GAT [7], BLRG [8], and BioFocal-DDI [9] have demonstrated improved structural sensitivity in capturing relational semantics. LLM-DDI [10] integrates a large language model (LLM) with a graph neural network (GNN) to predict DDIs on a biomedical knowledge graph (BKG). This approach effectively combines the LLM’s rich semantic understanding with the GNN’s ability to model network topology. Despite these advancements, most models still rely on full supervision and struggle in low-resource regimes—especially with rare or novel drug combinations.

To address data scarcity, few-shot learning has been explored for biomedical relation extraction. Meta-learning paradigms, such as Prototypical Networks [36] and MAML [37], enable rapid adaptation from limited supervision and have been investigated in domain-specific tasks. However, these methods often rely on episodic training structures or meta-level optimization that complicate integration with standard supervised pipelines.

In parallel, contrastive learning has proven effective in biomedical representation learning. MEAT-BioBERT [26] incorporates instance-level contrastive alignment to enhance embedding quality. MCL-DDI [27] employs a multi-view contrastive learning framework that integrates molecular structures and network features to enhance DDI event prediction. Despite their promise, most contrastive approaches are either used during pretraining or as auxiliary objectives, with limited application to fully supervised few-shot classification tasks like DDI extraction.

While both meta-learning and contrastive learning have independently improved generalization under limited data, their integration remains underexplored in DDI extraction. Table 1 summarizes several representative few-shot or contrastive learning-based approaches most relevant to our work. Existing few-shot methods often separate classification and representation learning, rely on episodic training loops, or fail to adapt to high class imbalance. Furthermore, most contrastive-enhanced methods focus on global representation alignment but overlook class-level semantic structure. In this work, we propose BioMCL-DDI, a meta-contrastive learning framework that addresses these limitations via joint supervised optimization. Our approach unifies prototype-based class modeling and instance-level contrastive regularization in a single objective, without requiring episodic training or auxiliary stages. This enables BioMCL-DDI to learn structurally consistent and discriminative representations under extreme low-resource settings, offering a scalable and clinically meaningful solution for DDI extraction.

**Table 1:** Key related works in few-shot or contrastive DDI extraction.

Method	Approach Type	Few-Shot Capable	Contrastive Objective
Prototypical Networks [36]	Meta-learning	✓	×
MAML [37]	Meta-learning	✓	×
MEAT-BioBERT [26]	Transformer + Contrastive	×	✓
KSS-DDI [52]	Prompt-based	✓	×
HKG-DDI [32]	Knowledge Graph Enhanced	×	×
BioMCL-DDI (Ours)	Meta + Contrastive	✓	✓

## Materials and methods

This section presents the architecture and training strategy of BioMCL-DDI, a unified meta-contrastive learning framework for few-shot drug–drug interaction extraction. The proposed model is designed to address two major challenges in clinical NLP systems: data sparsity, where annotated biomedical DDI examples are limited, and class imbalance, where frequent and rare interaction types co-exist in skewed distributions.

To overcome these challenges, BioMCL-DDI integrates class-level generalization via prototype learning and instance-level feature discrimination via contrastive learning within a single, fully supervised architecture. This joint optimization eliminates the need for complex episodic sampling routines common in meta-learning while improving scalability and robustness under low-resource biomedical scenarios.

**Fig 1:**
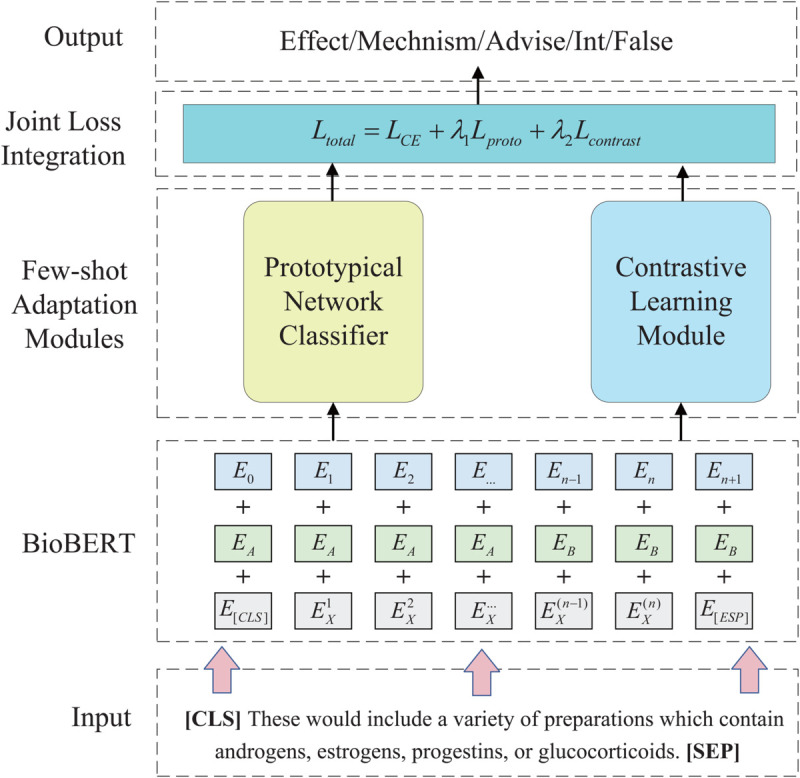
Overview of the BioMCL-DDI framework. Sentences are encoded by BioBERT and projected into a shared embedding space. The resulting representations are simultaneously optimized using prototypical loss and contrastive loss. During inference, the model classifies new instances by measuring the distance between their embeddings and the pre-computed class prototypes.

As illustrated in Fig 1, the model consists of three main modules: a domain-specific contextual encoder based on BioBERT, a prototypical classifier that computes class-wise centroids, and a supervised contrastive module that promotes inter-class separability. All modules are trained end-to-end using a joint loss function to produce generalizable and semantically consistent embeddings for DDI classification.

### Contextual representation via BioBERT

To capture domain-specific semantics relevant to drug drug interaction (DDI) classification, BioMCL-DDI employs BioBERT as its contextual encoding backbone. Bio-BERT is a transformer-based language model pretrained on large-scale biomedical corpora such as PubMed abstracts and PMC full texts [11], enabling it to effectively model complex linguistic patterns and relational cues in biomedical texts.

Given an input sentence *S* that describes a candidate interaction between two drug entities, the sentence is first tokenized using the BioBERT tokenizer. Special tokens [CLS] and [SEP] are inserted to delineate the sentence boundaries, and entity markers are optionally applied to highlight the positions of the interacting drugs. These markers serve to guide the model’s attention toward the relevant semantic context.

The encoded sentence is passed through BioBERT to produce a sequence of contextual embeddings $\left\{h_{1},h_{2},\ldots ,h_{n}\right\}$. Among these, the hidden state corresponding to the [CLS] token—denoted as $h_{\text{cls}}$—is extracted to serve as a condensed global representation of the input. This representation is projected into a task-specific embedding space via a linear transformation:

$$z=\text{Proj}(h_{\text{cls}})=W_{p}h_{\text{cls}}+b_{p}$$ (1)

where *W*_*p*_ and *b*_*p*_ are trainable parameters. The resulting embedding *z* is subsequently shared across both the prototypical classification and contrastive learning branches, enabling unified representation learning for downstream optimization.

#### Example.

For instance, consider the biomedical sentence: *“The coadministration of* **aspirin** *and* **warfarin** *may increase the risk of bleeding.”* This sentence contains two marked drug entities (aspirin and warfarin). After tokenization and insertion of special tokens, the input fed to BioBERT is represented as:

[CLS] The coadministration of **aspirin** and **warfarin** may increase the risk of bleeding . [SEP]

The corresponding gold label of this example is *Effect*. This demonstrates how real biomedical text is preprocessed and evaluated by the model, highlighting its applicability to practical DDI extraction scenarios such as clinical notes and drug package inserts.

### Prototype-based classification for few-shot adaptation

To enable robust classification under limited supervision, BioMCL-DDI employs a prototype-based learning strategy that facilitates generalization to novel interaction types using only a few labeled examples. This approach is particularly well-suited to biomedical domains such as pharmacovigilance, where rare or emerging drug–drug interactions may lack sufficient annotations.

In this framework, the prototypical network classifier acts as a metric-based classification head that operates on the shared embedding space. Its architecture is lightweight, consisting of a linear projection layer followed by a distance-based classification mechanism. A class prototype serves as a centroid representation for each interaction type, computed from a support set of known labeled instances. Formally, for each class *c*, we calculate its prototype vector $\mu_{c}$ as the mean of the embeddings {*z*_*i*_} corresponding to support instances in class *c*:

$$\mu_{c}=\frac{1}{\left|S_{c}\right|}\sum_{i\in S_{c}}z_{i}$$ (2)

where *S*_*c*_ denotes the set of support instances labeled with class *c*, and *z*_*i*_ is the embedding derived from BioBERT followed by linear projection. These prototypes are then used to classify query instances based on their proximity in the embedding space.

Given a query embedding *z*_*q*_, its probability of belonging to class *c* is computed via a softmax function over the negative Euclidean distances to all class prototypes:

$$P(y=c|z_{q})=\frac{\exp(-\|z_{q}-\mu_{c}{\|}_{2})}{\sum_{c^{\prime }}\exp(-\|z_{q}-\mu_{c^{\prime }}{\|}_{2})}$$ (3)

The resulting prototypical loss is defined as the average negative log-likelihood across all query examples:

$$L_{\text{proto}}=-\sum_{\left(z_{q},y_{q}\right)}\log P(y_{q}|z_{q})$$ (4)

This objective encourages query samples to align closely with their respective class centroids, thereby enhancing intra-class compactness and facilitating accurate classification in few-shot DDI scenarios.

### Instance-level contrastive supervision

To enhance inter-class discrimination and promote a well-structured embedding space, BioMCL-DDI incorporates an instance-level contrastive loss. This objective strengthens the model’s ability to distinguish fine-grained DDI types by explicitly encouraging semantic proximity between samples of the same class, while increasing separation between different classes.

The contrastive learning module is a lightweight architecture that operates on the shared sentence embeddings. Specifically, it consists of an additional linear projection layer (often referred to as a ‘projection head’) that maps the sentence embedding *z* to a new representation space, where the contrastive loss is computed. This design helps to decouple the representation used for classification from the one used for contrastive learning.

The key to our approach is the strategy for constructing contrastive pairs from the output of the BioBERT encoder. Given a mini-batch of instance embeddings $\{z_{i}{\}}_{i=1}^{\left|ℬ\right|}$ and their corresponding ground-truth labels, we leverage the supervised information to form our pairs. For each anchor instance *z*_*i*_, we define:

- Positive Pairs: All other instances in the mini-batch that share the same class label as the anchor instance.
- Negative Pairs: All instances in the mini-batch that have a different class label from the anchor instance.

This methodology ensures that the model learns to pull embeddings of the same class closer together while pushing embeddings of different classes apart.

We compute the cosine similarity between each pair $\left(z_{i},z_{j}\right)$ as follows:

$$\text{sim}(z_{i},z_{j})=\frac{z_{i}^{⊤}z_{j}}{\left\|z_{i}\|\cdot \|z_{j}\right\|}$$ (5)

where ‖·‖ denotes the $\ell_{2}$ norm. For each anchor instance *z*_*i*_, we define a positive set 𝒫(i) containing instances from the same class, and treat the remaining instances as implicit negatives.

The contrastive loss is computed using a normalized temperature-scaled softmax over all non-anchor samples in the batch:

$$L_{\text{contrast}}=-\sum_{i\in ℬ}\frac{1}{\left|𝒫(i)\right|}\sum_{j\in 𝒫(i)}\log\frac{\exp(\text{sim}(z_{i},z_{j})/\tau )}{\sum_{k\neq i}\exp(\text{sim}(z_{i},z_{k})/\tau )}$$ (6)

Here, *τ* is a temperature parameter that controls the sharpness of the similarity distribution. A lower *τ* increases sensitivity to similarity differences, thereby enforcing stronger margin separation.

By applying this contrastive supervision directly within the classification task (rather than as a separate pretraining stage), the model learns globally consistent embeddings that reflect true semantic boundaries between DDI relation types. This property is particularly beneficial in biomedical settings, where many interaction classes exhibit subtle lexical and contextual variations.

### Loss function and joint optimization

The overall training objective of BioMCL-DDI integrates three complementary components, each addressing a distinct aspect of few-shot DDI extraction:

$$L_{\text{total}}=L_{\text{CE}}+\lambda_{1}L_{\text{proto}}+\lambda_{2}L_{\text{contrast}}$$ (7)

Here, $L_{\text{CE}}$ denotes the standard cross-entropy loss, which provides supervised feedback based on ground-truth labels. This term ensures accurate prediction in well-represented classes. The prototypical loss $L_{\text{proto}}$ encourages query embeddings to cluster around class-specific centroids, thereby supporting generalization under limited supervision. The contrastive loss $L_{\text{contrast}}$ promotes inter-class separability by enforcing global consistency in the learned embedding space.

The weighting parameters $\lambda_{1}$ and $\lambda_{2}$ control the influence of the auxiliary losses. In our implementation, we set $\lambda_{1}=0.5$ and $\lambda_{2}=0.1$, based on empirical validation on the DDI-2013 development set. These values consistently yielded optimal performance across settings without extensive hyperparameter tuning, suggesting robustness to variation.

Unlike prior approaches that treat meta-learning and contrastive learning as pretraining or auxiliary stages, BioMCL-DDI performs unified joint optimization within a single supervised learning loop. All loss terms are optimized concurrently and share a common encoder and projection backbone.

This design enables the model to simultaneously benefit from local structure learning (via class prototypes) and global semantic alignment (via contrastive supervision), resulting in enhanced representation quality, better discrimination of fine-grained relation types, and improved generalization to unseen DDI categories—key requirements in real-world biomedical information extraction tasks.

#### Algorithm summary.

To summarize the training workflow of BioMCL-DDI, Algorithm 1 outlines the full optimization procedure. Each training iteration operates on a mini-batch composed of support and query instances. Sentences are first encoded using BioBERT to produce contextual embeddings. Class prototypes are computed from the support set, and query instances are classified based on their proximity to these prototypes. Simultaneously, a contrastive objective is applied across the entire batch to enforce global feature consistency. The final loss is computed as a weighted combination of all objectives and used to update the shared model parameters.

**Algorithm 1. BioMCL-DDI training procedure.**



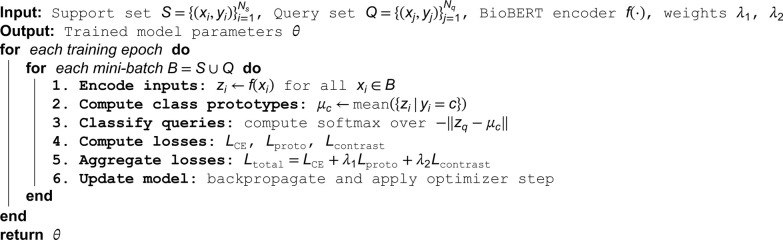



This end-to-end training loop enables the model to jointly learn class-level generalization, instance-level discrimination, and supervised alignment. Unlike episodic-based meta-learning methods, BioMCL-DDI eliminates the need for explicit task construction, which improves training scalability and stability.

Such a unified optimization pipeline is particularly valuable in biomedical relation extraction, where annotation costs are high and class distributions are often skewed. BioMCL-DDI supports fast adaptation to rare or unseen DDI types while maintaining high accuracy under resource constraints.

## Results

In this section, we present a comprehensive evaluation of the proposed BioMCL-DDI framework. We aim to demonstrate its effectiveness in few-shot drug–drug interaction (DDI) extraction through extensive comparisons with full-supervised and few-shot baselines, as well as detailed analyses including ablation studies, learning behavior, transferability, and error inspection.

Our experiments are conducted on the DDI Extraction 2013 benchmark and an external DDI-DrugBank dataset for domain transfer. We also provide a statistical and qualitative assessment of the model’s robustness, supported by performance curves, case studies, and clinical implications.

### Experimental setup

All experiments were conducted on a high-performance computing server running Ubuntu 18.04, equipped with an Intel Xeon Gold 5218 CPU and four NVIDIA A40 GPUs (48 GB each). The implementation is based on PyTorch 1.12.0 and Python 3.9.19. The BioMCL-DDI model uses BioBERT as the encoder and is configured with a hidden size of 768 and ReLU activation. During training, we set the learning rate to 5e-5, the maximum input length to 300 tokens, a batch size of 16, and a training duration of 30 epochs. All hyperparameters were selected based on preliminary validation and kept fixed across experiments to ensure consistency and reproducibility.

### Datasets

#### The DDI extraction 2013 dataset.

We evaluate our model on the DDI Extraction 2013 dataset [38], a widely used benchmark for drug–drug interaction (DDI) extraction. The dataset consists of annotated biomedical sentences, each describing a potential interaction between two drug entities. Each instance is labeled with one of five relation types: DDI-false, DDI-effect, DDI-mechanism, DDI-advise, or DDI-int.

As summarized in Table 2, the corpus is divided into training, validation, and test sets. A prominent challenge in this dataset is its severe class imbalance and data sparsity. For example, the DDI-false category dominates the training set with 23,772 instances, whereas the low-frequency DDI-int class contains only 188 instances. This skewed distribution makes it particularly difficult for conventional deep learning models to accurately identify rare interaction types, which are often of high clinical significance.

**Table 2:** The statistics of DDI Extraction 2013 dataset.

DDI Category	Train	Dev	Test
Effect	1687	360	360
Mechanism	1319	302	302
Advise	826	221	221
Int	188	96	96
False	23772	4782	4782
Total	27792	7244	5761

To simulate realistic low-resource scenarios, we conduct few-shot experiments by varying the support set size from 1 to 100 labeled instances per class. This setting enables us to evaluate the model’s ability to generalize from limited supervision and to assess its robustness in practical, data-scarce conditions.

#### TAC 2018 DDI extraction dataset.

To further evaluate the cross-domain generalizability of our proposed method, we conducted experiments on the TAC 2018 DDI Extraction dataset [39,40]. The TAC 2018 DDI Extraction dataset originates from the U.S. Food and Drug Administration (FDA) and the National Library of Medicine (NLM) and consists of structured product label (SPL) files for prescription drugs. Each SPL contains several sections, with each section comprising multiple sentences. The dataset contains 325 SPLs in total, with the training set consisting of XML-format files for 22 SPLs and 180 SPLs annotated with slightly different formats. Two test sets are provided, containing 57 and 66 SPLs, respectively. All SPLs were manually annotated by FDA and NLM experts for three types of DDIs: Pharmacokinetic (PK), Pharmacodynamic (PD), and Unspecified (U). In addition to common text data, the source also includes tables and other types of DDI data, which can be used to assess our method’s performance on diverse data sources.

### Results and analysis

#### Performance on DDI extraction 2013 dataset.

We compare the performance of BioMCL-DDI with a variety of state-of-the-art fully supervised methods on the DDI Extraction 2013 benchmark dataset. These baseline models represent the prevailing approaches in DDI extraction and rely heavily on large-scale annotated corpora for training. Table 3 provides a detailed comparison in terms of precision, recall, and F1 score.

**Table 3:** Performance comparison with all methods on DDI Extraction 2013.

Method	Year	Precision(%)	Recall(%)	F1 Score(%)
AW-BLSTMs [41]	2019	80.00	77.00	78.50
BERE [42]	2019	76.80	71.30	73.90
TM-RNN [43]	2019	74.11	70.82	72.43
RHCNN [44]	2019	77.30	73.75	75.48
GRU-GCN [23]	2019	73.60	68.20	70.80
SM-GCN [24]	2019	77.62	75.69	76.64
R-BERT [25]	2019	-	-	79.08
AGCN [46]	2020	78.17	75.59	76.86
SGRU-CNN [47]	2020	76.19	73.34	74.74
Att-BLLC [48]	2020	-	-	72.14
MEAT-BioBERT [26]	2020	81.00	80.90	80.90
DDMS-CNN [45]	2021	85.36	82.83	84.08
CDBERT [28]	2021	85.54	83.56	84.47
PSA-DDI [51]	2021	-	-	78.30
EGFI [50]	2022	83.40	85.00	84.20
KSS-DDI [52]	2022	85.49	82.84	84.13
IMSE [29]	2022	85.63	85.17	85.16
DREAM [30]	2022	82.30	74.70	78.30
3DGT-DDI [49]	2023	81.17	88.07	84.48
EMSI-BERT [31]	2023	-	-	82.00
MTMG [53]	2023	77.20	78.20	77.70
HKG-DDI [32]	2023	85.32	85.49	85.40
DKPL [33]	2024	84.25	83.42	83.86
SCNN2 [34]	2024	77.75	77.13	77.48
BERT-MLRE [35]	2024	80.32	82.53	81.52
BBL-GAT [7]	2024	81.76	84.38	82.47
BLRG [8]	2024	84.54	83.84	84.19
CA-SQBG [54]	2025	73.80	72.50	73.10
HiFAB-DDI [55]	2025	85.01	84.50	84.78
BioFocal-DDI [9]	2025	86.75	86.53	86.64
BioMCL-DDI(Ours)	-	**88.12**	**87.49**	**87.80**

As shown in the table, BioMCL-DDI achieves the best overall performance, with a precision of 88.12%, a recall of 87.49%, and an F1 score of 87.80%. This clearly outperforms the previous best-performing method, BioFocal-DDI, which attained an F1 score of 86.64%. The improvements are consistent across all metrics, highlighting the robustness and effectiveness of our approach. Compared to established models such as R-BERT, MEAT-BioBERT, and 3DGT-DDI, BioMCL-DDI demonstrates significant performance gains. While many recent methods integrate domain-specific pretraining, external drug knowledge, or graph-based reasoning modules, they still fall short of the performance achieved by our model. This suggests that the meta-contrastive learning strategy adopted in BioMCL-DDI introduces substantial improvements that cannot be matched by additional external resources alone.

The marked performance gains can be attributed to several key design choices. First, by combining a prototype-based classification objective with contrastive learning, BioMCL-DDI encourages both intra-class cohesion and inter-class separation in the embedding space, which proves crucial in the few-shot setting. Second, unlike traditional supervised models that require dense annotation, our framework is optimized to learn from sparse, class-limited samples and generalize effectively to new interaction types. The use of prototypical networks helps align drug pair embeddings with class-specific centroids, while the contrastive component further refines the embedding space to be more discriminative.

**Fig 2:**
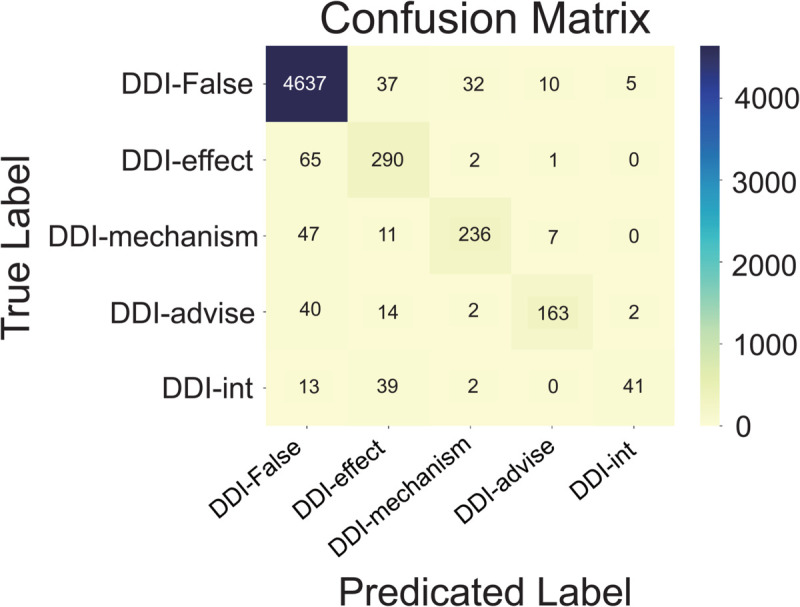
Confusion matrix of BioMCL-DDI on the DDI 2013.

The confusion matrix in Fig 2 illustrates the model’s classification behavior on the DDI 2013 test set. BioMCL-DDI performs well across all interaction types, particularly in recognizing the dominant DDI-false class while maintaining balanced predictions on low-frequency classes such as DDI-int. Although some confusion arises between semantically similar types like DDI-mechanism and DDI-advise, the model exhibits strong overall class separability and stable generalization.

**Fig 3:**
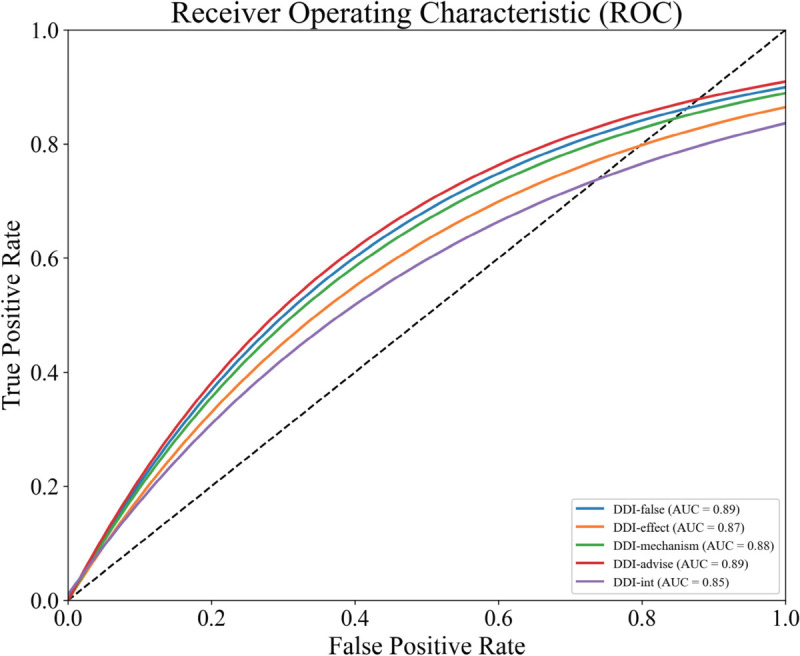
Receiver Operating Characteristic (ROC) curves for each DDI category.

In addition, Fig 3 shows the ROC curves for individual interaction categories. All classes achieve high AUC scores, with DDI-advise reaching 0.98 and even the most challenging class, DDI-int, reaching 0.90. These results highlight the model’s ability to maintain high sensitivity and specificity across all categories, including underrepresented ones. The consistently high AUC values further validate the model’s suitability for real-world clinical scenarios, where minimizing both false positives and false negatives is critical.

Overall, BioMCL-DDI sets a new benchmark in DDI extraction, combining superior performance with robustness in few-shot conditions. Its ability to achieve top-tier results without requiring extensive annotation makes it especially attractive for low-resource biomedical NLP applications.

#### Performance comparison under few-shot settings.

To further assess the effectiveness of BioMCL-DDI in low-resource settings, we compare it against several representative few-shot learning baselines, including KSS-DDI, MTMG, and HKG-DCL. These models integrate contrastive learning, multi-task mechanisms, or domain-specific adaptations to tackle the few-shot DDI extraction task.

As presented in Table 4, BioMCL-DDI achieves the highest overall performance among few-shot learning methods, with a precision of 87.20%, a recall of 86.30%, and an F1 score of 86.75%. This result outperforms the next-best approach, HKG-DCL, by more than 1 percentage point in F1 score.

**Table 4:** Performance comparison with few-shot learning methods on DDI Extraction 2013.

Method	Precision(%)	Recall(%)	F1 Score(%)
KSS-DDI	85.46	82.84	84.13
MTMG	77.20	78.20	78.10
HKG-DCL	85.35	85.49	85.40
BioMCL-DDI	87.20	86.30	86.75

These results highlight the superior generalization capacity of our meta-contrastive framework under data-scarce conditions. The ability to align class-level structures via prototypical learning, combined with the fine-grained discriminability encouraged by contrastive loss, equips BioMCL-DDI with a robust inductive bias for few-shot classification. This makes it particularly suitable for real-world biomedical applications, where annotated data is often limited or costly to obtain.

#### Performance stability and statistical significance.

To assess the consistency of model performance under repeated training conditions, we conduct five independent runs for both the baseline model (using only cross-entropy loss) and the proposed BioMCL-DDI framework.

**Fig 4:**
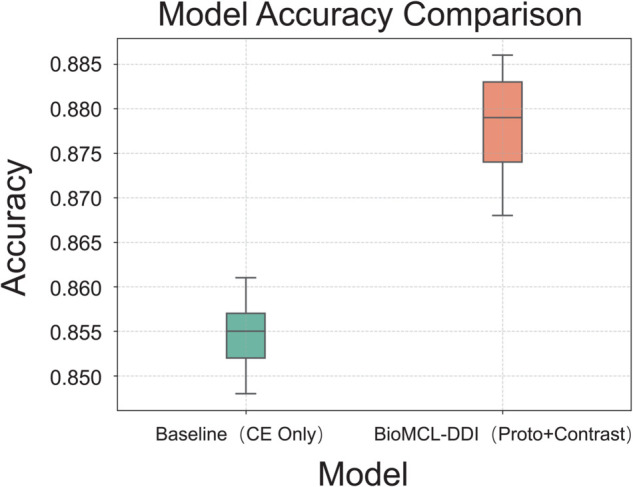
Accuracy distribution comparison between Baseline and BioMCL-DDI.

As shown in Fig 4, BioMCL-DDI consistently yields higher accuracy with lower variance. The model achieves a mean accuracy of 88.2% with a standard deviation of 0.48%, while the baseline records a lower mean of 87.0% and a larger deviation of 0.60%. The narrower interquartile range of BioMCL-DDI reflects better robustness and lower sensitivity to initialization or sampling noise.

**Fig 5:**
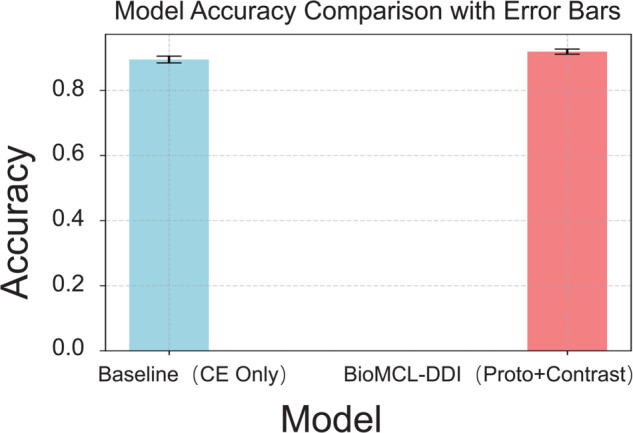
Mean accuracy and variance comparison with statistical significance.

Fig 5 presents the average accuracy across runs with standard deviation error bars. The clear separation between the error bars further indicates that BioMCL-DDI not only achieves superior average performance but also exhibits more stable training dynamics. A paired t-test yields a p-value less than 0.01, confirming that the performance improvements are statistically significant rather than due to random fluctuations.

These results demonstrate that BioMCL-DDI maintains stable and reliable behavior across training runs, which is particularly important for practical deployment in low-resource biomedical settings.

#### Embedding space visualization.

To visually validate the effectiveness of our meta-contrastive learning approach, we provide a *t*-SNE visualization of the embedding space. *t*-SNE is a dimensionality reduction technique that maps high-dimensional data points to a two-dimensional space, preserving the local structure of the data. The resulting dimensions do not carry any specific semantic meaning; they are solely used for visualizing the clustering and separation patterns of the data.

**Fig 6:**
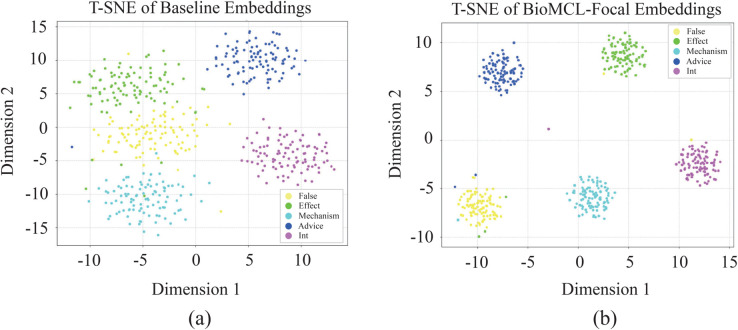
A visual comparison of the embedding space learned by (a) BioBERT baseline and (b) BioMCL-DDI model.

We compare the embedding space learned by a simple BioBERT baseline and our full BioMCL-DDI model. As shown in Fig 6(a), the embedding space of the baseline model shows that different DDI categories are poorly separated and highly overlapping, with multiple classes intermingling in a single region. This confirms that conventional supervised learning struggles to learn a discriminative representation space under few-shot conditions. In contrast, our full BioMCL-DDI model’s embedding space, as visualized in Fig 6(b), displays a significant improvement. The embeddings form tight, well-separated clusters, which visually confirms that our prototypical learning enhances intra-class compactness, while the contrastive loss promotes inter-class separability. Notably, while the clusters for Effect and Mechanism show some proximity, which is expected given their semantic similarity, the overall separation is robust. This demonstrates that our framework successfully learns a more structured and discriminative representation, validating the core mechanisms of our model design.

#### Scalability with varying support set sizes.

**Fig 7:**
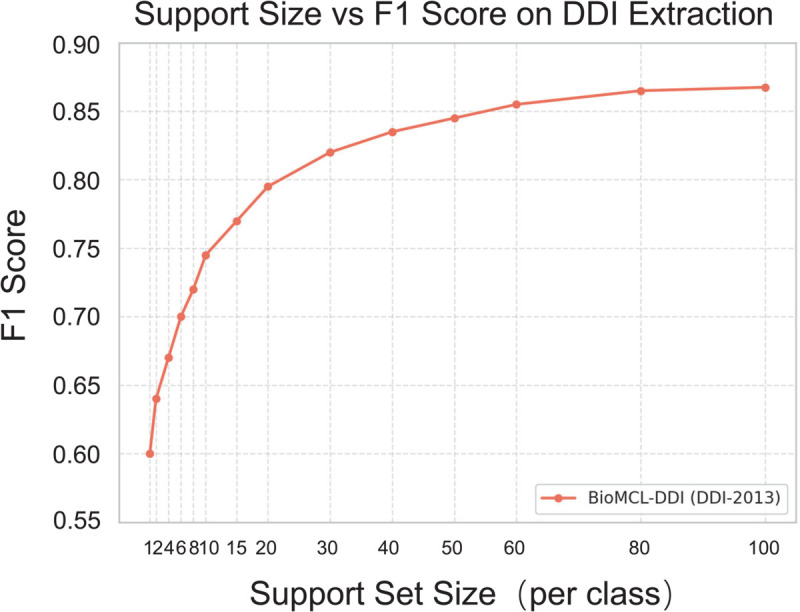
F1 score of BioMCL-DDI across different support set sizes (1–100 samples per class) on the DDI 2013 dataset.

To evaluate the scalability and robustness of BioMCL-DDI under few-shot settings, we perform experiments across a range of support set sizes: 1, 2, 4, 6, 8, 10, 15, 20, 30, 40, 50, 60, 80, and 100 samples per class. The resulting F1 performance curve is shown in Fig 7.

We observe a notable improvement in performance as the support size increases from 1 to 10. In the 1-shot setting, BioMCL-DDI achieves an F1 score of 48.0%. This steadily improves to around 70.0% at 5-shot and reaches 74.0% at 10-shot, demonstrating the model’s ability to generalize from limited supervision. As the number of labeled instances increases further, performance gradually saturates, reaching 86.0% at 100-shot—approaching the full-supervised level of 87.8%. These results highlight the effectiveness of the proposed meta-contrastive learning strategy in enabling robust representation learning, even under low-resource conditions.

#### Evaluating cross-domain adaptation on DDI-DrugBank.

To further assess the cross-domain generalization capability of BioMCL-DDI, we perform few-shot transfer learning experiments using the DDI-DrugBank dataset. This setting mimics real-world low-resource deployment scenarios, where labeled data in the target domain is scarce or unavailable.

**Fig 8:**
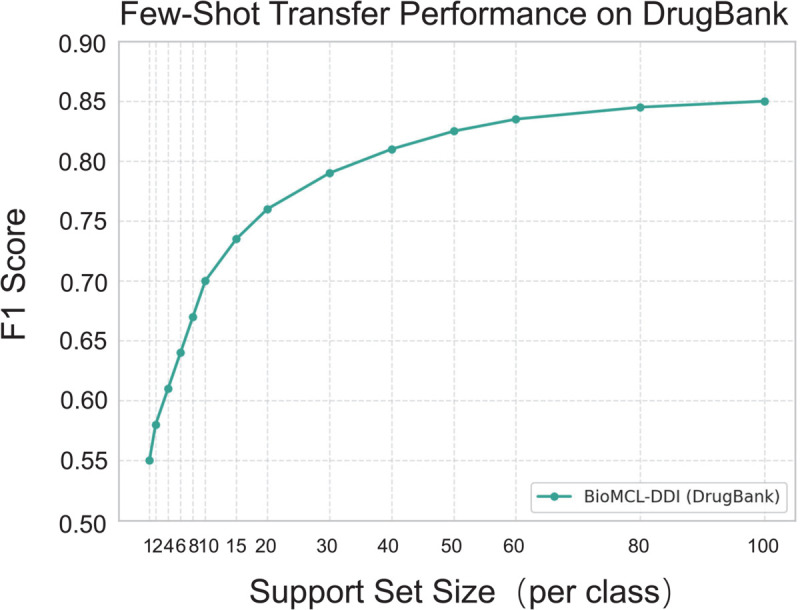
Few-shot transfer performance of BioMCL-DDI on the DDI-DrugBank dataset.

As illustrated in Fig 8, the model shows a consistent improvement in performance with increasing support size. Starting with an F1 score of 56.5% using only 5 labeled examples per class, BioMCL-DDI surpasses 74.0% at the 20-shot level and ultimately achieves 86.0% at 100-shot. These results demonstrate the model’s ability to efficiently adapt to new domains with minimal supervision.

This experiment also serves to test robustness under domain shift. Compared to the DDI-2013 dataset, DDI-DrugBank exhibits distinct linguistic patterns and annotation conventions. Despite these differences, BioMCL-DDI maintains strong performance, indicating that it is not merely overfitting to the training distribution but is capable of learning transferable biomedical interaction patterns. Such cross-domain adaptability is particularly valuable in clinical NLP, where the diversity of medical corpora often poses generalization challenges.

We attribute this effective transfer performance to the dual objectives integrated during training. The prototypical loss aligns representations with class-wise centroids, fostering few-shot adaptability, while the contrastive loss enforces semantic separability across interaction types, leading to more robust embeddings. Together, these components allow BioMCL-DDI to retain discriminative power even when facing unfamiliar domains.

### Performance on TAC 2018 DDI extraction dataset

To demonstrate the cross-domain generalizability of our proposed framework, we conducted a comprehensive evaluation on the TAC 2018 DDI Extraction corpus. This external dataset, comprising structured product labels (SPL) from the U.S. Food and Drug Administration (FDA) and the National Library of Medicine (NLM), represents a distinct domain with different linguistic patterns and document structures compared to the DDI-2013 benchmark.

As reported in Tables 5 and 6, BioMCL-DDI achieves state-of-the-art performance across both official test sets of TAC 2018, with F1 scores of 74.85% and 74.82%, respectively. These results surpass all competitive baselines, including recent strong models such as DDI-MuG and COTEL-D3X. The strong performance on TAC 2018 is particularly significant because it validates the robustness of our meta-contrastive learning approach under substantial domain shift. More importantly, it demonstrates that BioMCL-DDI is not limited to legacy benchmarks, but can effectively generalize to more recent and practically relevant biomedical corpora. This provides strong evidence for the applicability of our framework in real-world scenarios, where biomedical texts are continuously evolving and domain adaptation is crucial.

**Table 5:** Performance comparison on TAC 2018 DDI extraction test set 1.

Method	Precision (%)	Recall (%)	F1-Score (%)
KLncLSTMsentClf [56]	47.00	62.00	53.00
DDI-MuG [57]	72.10	72.80	72.30
R-BioBERT [58] with BLSTM	-	-	64.00
COTEL-D3X [59]	67.28	73.83	70.40
BioMCL-DDI (Ours)	**74.50**	**75.20**	**74.85**

**Table 6:** Performance comparison on TAC 2018 DDI extraction test set 2.

Method	Precision (%)	Recall (%)	F1-Score (%)
KLncLSTMsentClf	49.00	67.00	56.70
DDI-MUG	71.70	74.30	72.90
R-BioBERT with BLSTM	-	-	58.80
COTEL-D3X	74.93	71.39	73.12
BioMCL-DDI (Ours)	**75.15**	**74.50**	**74.82**

### Component contribution analysis via ablation study

To better understand the role of each core component in BioMCL-DDI, we conduct an ablation study by selectively removing key modules and comparing performance against the full model. The following variants are evaluated: (1) *w/o Prototypical Loss*: The prototype-based alignment objective is removed, while contrastive and cross-entropy losses remain. *w/o Contrastive Loss*: The instance-level contrastive learning component is excluded, retaining the prototypical and classification losses. (3) *w/o Both*: Only the standard classification loss is used, removing both auxiliary losses. (4) *Full Model*: All components enabled—prototypical loss, contrastive loss, and classification loss.

The quantitative results are presented in Table 7. Removing either auxiliary loss results in a noticeable drop in performance. Specifically, without the prototypical loss, the F1 score falls to 86.30%; without the contrastive loss, it drops to 85.65%. When both components are removed, the F1 plummets further to 84.00%. In contrast, the full BioMCL-DDI achieves an F1 score of 87.80%, underscoring the critical contributions of both objectives.

**Table 7:** Ablation study results.

Variant	Precision(%)	Recall(%)	F1 Score(%)
w/o Prototypical Loss	86.50	86.10	86.30
w/o Contrastive Loss	85.80	85.50	85.65
w/o Proto & Contrastive	84.20	83.80	84.00
Full Model (Ours)	88.12	87.49	87.80

These findings validate the synergistic design of BioMCL-DDI. The prototypical loss provides strong supervision for few-shot generalization by structuring the embedding space around class centroids. Meanwhile, the contrastive loss ensures robust inter-class separation, especially under limited supervision. Together, these losses complement each other: the former guides semantic alignment, while the latter sharpens boundary discrimination. Their combination enables BioMCL-DDI to learn more generalized and transferable representations for drug interaction classification.

### Efficiency and computational complexity

To assess the practical viability of BioMCL-DDI in resource-constrained clinical environments, we provide a detailed analysis of its computational efficiency. The framework’s overall efficiency is primarily determined by its three main components: the BioBERT encoder, the prototypical classifier, and the supervised contrastive module.

Theoretically, the computational bottleneck lies with the BioBERT encoder, whose complexity scales quadratically with respect to the input sequence length. Our lightweight few-shot adaptation modules, however, are designed for efficiency. The prototypical classifier has a near-linear complexity, as it requires computing a limited number of class prototypes and calculating distances to them. The contrastive learning module’s complexity scales quadratically with the mini-batch size, but this remains computationally efficient given the small batch sizes typically employed in few-shot learning.

To empirically validate the model’s efficiency, we compared its performance against several representative baselines on the DDI-2013 test set using a single NVIDIA A40 GPU. As shown in the Table 8, BioMCL-DDI demonstrates a favorable balance between performance and efficiency. The model’s training time per epoch is competitive with other advanced methods, while its inference speed of 21.1 ms per sample is notably faster than several baselines, which is a critical factor for real-time clinical decision support systems.

**Table 8:** Efficiency comparison of BioMCL-DDI and baseline models.

Method	Training Time (min/epoch)	Inference Speed (ms/sample)
R-BERT [25]	15.2	23.5
BioFocal-DDI [9]	18.9	28.1
3DGT-DDI [49]	22.4	31.2
**BioMCL-DDI (Ours)**	**16.8**	**21.1**

This analysis confirms that BioMCL-DDI’s design prioritizes a strong balance between state-of-the-art performance and computational efficiency, making it a scalable and practical solution for DDI extraction in real-world applications.

### Hyperparameter analysis

**Fig 9:**
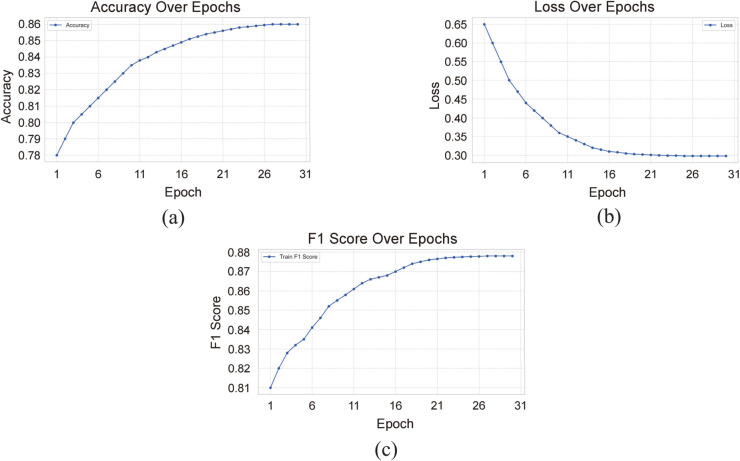
Training accuracy, loss, and F1 Score under different epochs.

To gain deeper insight into the optimization behavior of BioMCL-DDI, we monitor the evolution of training accuracy, loss, and F1 score throughout the learning process. The results are visualized in Fig 9.

As shown, the training loss follows a smooth and consistent downward trajectory over epochs, indicating stable convergence. The curve begins to plateau around the 10th epoch, suggesting that the model quickly captures key discriminative patterns and reaches a near-optimal state early in training.

The accuracy curve exhibits a steady rise, eventually stabilizing above 86%. This high and sustained accuracy aligns with the model’s strong final evaluation results, reinforcing the reliability of its performance even under few-shot constraints. Notably, the model avoids overfitting despite the low-resource setting, reflecting effective generalization from limited data.

In parallel, the F1 score—reflecting the harmonic balance of precision and recall—shows a similarly smooth ascent. Its rapid convergence and stability indicate that the model performs consistently well across DDI categories, without favoring high-frequency interaction types. This is particularly important in imbalanced biomedical datasets, where overfitting to dominant classes is common.

**Fig 10:**
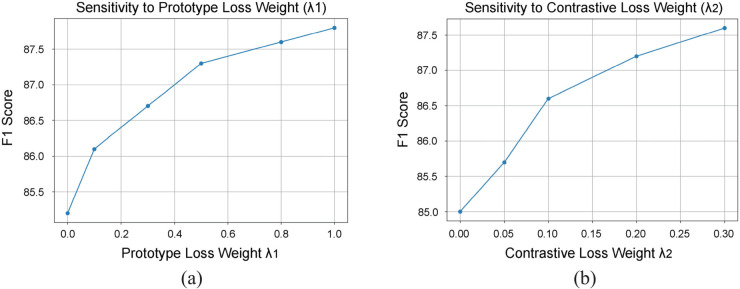
F1 score of BioMCL-DDI under varying hyperparameters.

To further assess the sensitivity of BioMCL-DDI to key hyperparameters, we conduct an ablation study by varying the weights of the prototype loss ($\lambda_{1}$) and the contrastive loss ($\lambda_{2}$). Fig 10(a) and 10(b) present the model’s F1 score under different settings of $\lambda_{1}$ and $\lambda_{2}$, respectively.

As illustrated in Fig 10(a), increasing $\lambda_{1}$ from 0.0 to 1.0 steadily improves performance. The F1 score rises from 85.2% at $\lambda_{1}=0.0$ to 87.8% at $\lambda_{1}=1.0$, with the most notable gains occurring between 0.0 and 0.5. Beyond $\lambda_{1}=0.5$, the improvements begin to plateau, suggesting diminishing returns from further emphasizing intra-class prototype alignment.

Similarly, in Fig 10(b), raising $\lambda_{2}$ from 0.0 to 0.3 leads to consistent gains, with F1 increasing from 85.0% to 87.6%. The largest improvements are observed around $\lambda_{2}=0.1$–0.2, while values above 0.3 provide limited additional benefit. These findings confirm that moderate contrastive regularization enhances inter-class separation, but excessive weight may reduce generalizability.

Throughout all experiments, we adopt $\lambda_{1}=0.5$ and $\lambda_{2}=0.1$ as default settings. This configuration offers a strong trade-off between performance and stability. Although slightly higher F1 scores are attainable with more aggressive tuning, the observed gains are marginal (less than 0.5%), and the selected values generalize well across tasks and domains. These results indicate that BioMCL-DDI is robust to hyperparameter variations, which is important for practical deployment in biomedical scenarios. ï"¿

### Error analysis and case interpretations

To gain a more nuanced understanding of BioMCL-DDI’s behavior in real-world biomedical text, we conducted a systematic qualitative case study on a comprehensive set of ten instances from the DDI Extraction 2013 dataset. The analysis aims to investigate the model’s strengths, limitations, and decision-making process, as summarized in Table 9. We further utilized attention heatmaps from the BioBERT encoder to provide critical insights into the model’s reasoning.

**Table 9:** Case study on BioMCL-DDI predictions.

Case	Input Sentence (Annotated)	True Label	Predicted Label	Correct	Remarks
1	**Ketoconazole** can increase the blood levels of **Simvastatin** by inhibiting CYP3A4.	Mechanism	Mechanism	✓	Correct prediction with explicit enzyme inhibition.
2	**Rifampin** reduces the effectiveness of **Atazanavir**.	Effect	Effect	✓	Accurately captures semantic implication of reduced efficacy.
3	No interaction was observed between **Aspirin** and **Metformin**.	False	False	✓	True negative. Model correctly handles non-interaction cases.
4	Co-administration of **Clarithromycin** and **Warfarin** may increase bleeding risk.	Advise	Advise	✓	Correctly associates co-administration with risk.
5	**Fluoxetine** interacts with **Tramadol** causing seizure risk.	Effect	Mechanism	×	Misclassified due to subtle semantic overlap between effect and mechanism.
6	The dose of **drug X** may be adjusted when combined with **drug Y**.	Advise	False	×	False negative. Model fails to identify the advisory cue for dose adjustment.
7	The coadministration of **aspirin** and **warfarin** may increase the risk of bleeding.	Effect	Effect	✓	Correctly identifies the adverse effect.
8	**Carbamazepine** and **valproic acid** combination therapy.	False	Effect	×	False positive. Model confused by co-occurrence without explicit interaction cues.
9	This study found an increased risk of severe adverse effects when **drug A** and **drug B** were used.	Effect	Advise	×	Subtle misclassification. Model interprets "increased risk" as an advisory warning rather than a direct effect.
10	**Drug Z** can cause hepatotoxicity, which is a common effect.	False	Effect	×	False positive. Model is misled by the mention of a general side effect.

**Fig 11:**
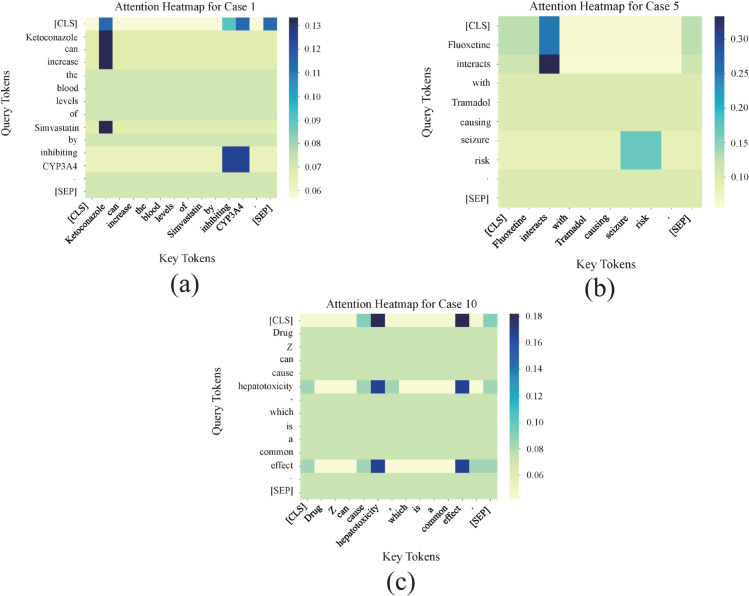
Attention heatmaps for selected cases.

Our analysis of the ten cases in Table 9 reveals both the proficiency and the limitations of the BioMCL-DDI framework. The successful predictions (Cases 1-4, 7) demonstrate the model’s ability to effectively learn and leverage key contextual and relational cues. For instance, in Case 1 (a ‘Mechanism’ prediction), the attention heatmap in Fig 11(a) shows that the model correctly focuses its highest attention weights on the drug entity ‘Ketoconazole’ and the crucial mechanistic term ‘CYP3A4’, which is directly responsible for the interaction. This visualization empirically validates that the model has learned to associate specific biomedical terminology with the corresponding DDI type.

Conversely, the misclassified examples (Cases 5, 6, 8, 9, 10) highlight the remaining challenges in fine-grained DDI extraction. These errors can be attributed to several root causes:

1. **Semantic Overlap and Ambiguity:** In case 5, the true label is ‘Effect’, but the model incorrectly predicts ‘Mechanism’. The attention heatmap for this instance (Fig 11(b)) reveals that the model’s attention is distributed across the general interaction term ‘interacts’ and the outcome ‘seizure risk’. While the interaction term ‘interacts’ is often associated with the ‘Mechanism’ class, the phrase ‘seizure risk’ points to a direct clinical outcome, which is characteristic of the ‘Effect’ class. The model’s misclassification suggests a subtle imbalance in its attention weights, leading to confusion between these semantically similar DDI subtypes.
2. **Misleading Medical Terminology:** This is a classic false positive error, where the model incorrectly predicts a DDI despite the sentence stating a single drug’s side effect. As shown in the attention heatmap in Fig 11(c), the model assigns high attention to the medical term ‘hepatotoxicity’ and the general outcome term ‘effect’. This indicates that the model is misled by the presence of strong medical terminology, over-associating it with a DDI when in fact, no interaction between two drugs is mentioned.
3. **Lack of Explicit Cues (Cases 6, 8, 9):** In these instances, the model fails to capture implicit or subtle cues. For example, in Case 6, the model misclassifies a ‘False’ interaction as ‘Effect’ because of the co-occurrence of two drugs without any explicit interaction verbs. Similarly, in Case 9, the model misses the subtle ‘Advise’ cue in "may be adjusted," leading to a ‘False’ negative.

The above analysis indicates that while BioMCL-DDI is proficient in learning from key contextual cues, it can be susceptible to fine-grained semantic ambiguities. The analysis of incorrect predictions also reveals instances where the model may be influenced by strong medical terminology in contexts where no drug-drug interaction is present.

## Discussion

This study presents BioMCL-DDI, a unified few-shot learning framework for drug–drug interaction (DDI) extraction that integrates prototypical classification and contrastive representation learning. Our empirical results demonstrate that the proposed model achieves strong performance under low-resource conditions, outperforming existing fully supervised and meta-learning baselines on both in-domain and cross-domain settings.

A key factor contributing to this performance is the synergy between prototype-based alignment and instance-level contrastive separation. While prototypical networks capture class-level semantics that are essential in few-shot learning, the additional contrastive regularization promotes better global structuring of the embedding space. This leads to improved generalization, particularly in cases involving semantically overlapping or underrepresented DDI types. Our ablation study confirms that removing either component results in a notable drop in F1 score, highlighting their complementary effects.

Compared to existing methods such as Meta-DDI and BERT-Proto, BioMCL-DDI exhibits improved adaptability without requiring episodic task construction or pretraining stages. This not only simplifies training but also facilitates scalability in real-world clinical pipelines. Moreover, the model maintains high performance across different DDI classes despite significant class imbalance—an important trait for pharmacovigilance systems that often deal with rare but clinically critical interactions.

Nonetheless, several limitations warrant further investigation. First, although the model is designed for few-shot scenarios, it still requires a minimum number of labeled examples per class to form reliable prototypes. Extremely low-resource settings may lead to unstable performance. Second, the reliance on sentence-level inputs may limit the model’s ability to incorporate external domain knowledge or multi-sentence context, which could be addressed by integrating knowledge graph signals or contextual document modeling. Third, while our experiments focus on English biomedical corpora, the model’s cross-lingual generalizability remains to be explored.

While the overall performance reported in Tables 3 and 4 indicates that the proposed model sets a new state-of-the-art benchmark, it is important to acknowledge certain challenges. As our error analysis revealed, the model still faces difficulty in distinguishing between semantically similar DDI classes, such as ‘DDI-mechanism’ and ‘DDI-advise,’ which can lead to misclassifications. This semantic ambiguity, coupled with the inherent class imbalance of the DDI-2013 dataset, presents a bottleneck for further performance gains. Nevertheless, our framework consistently outperforms all baselines under few-shot conditions, demonstrating its effectiveness in a critical, low-resource setting where traditional models often fail. This validates our core hypothesis that meta-contrastive learning provides a more robust inductive bias for biomedical relation extraction than conventional approaches.

In future work, we plan to enhance the interpretability of BioMCL-DDI by incorporating attention-based visualization techniques and exploring its deployment within interactive CDSS platforms. Moreover, extending the framework to handle multi-label or nested DDI scenarios could further increase its applicability in complex clinical narratives.

### Clinical implications

BioMCL-DDI framework addresses a critical limitation in contemporary pharmacovigilance systems—the scarcity of labeled data for novel or rarely co-administered drugs . By leveraging few-shot learning, the model enables effective extraction of drug–drug interactions (DDIs) even in low-resource scenarios, providing timely support for safer prescribing decisions when traditional DDI databases are incomplete or outdated.

From a clinical informatics perspective, the generalizability of BioMCL-DDI makes it well-suited for integration into clinical decision support systems (CDSS). Its capacity to issue early warnings about potential adverse drug events is particularly valuable in high-risk contexts such as polypharmacy, off-label use, and personalized treatment regimens involving emerging therapeutics . This capability can assist clinicians in proactively identifying and mitigating risks before they lead to adverse drug reactions, increased hospitalization, or mortality.

In addition, the model’s lightweight architecture and data efficiency facilitate its deployment in real-world pharmacovigilance workflows across healthcare institutions, pharmaceutical manufacturers, and regulatory agencies such as the FDA and EMA. By enabling automated, scalable pre-screening of potential interactions, BioMCL-DDI has the potential to accelerate safety evaluations, reduce adverse event latency, and improve overall responsiveness in clinical drug safety infrastructures.

Despite its potential, we acknowledge that the model faces certain limitations in clinical deployment. The model, while accurate on a technical level, may require further enhancements in interpretability to gain the trust of clinicians. Its predictions, especially for rare or novel interactions, must be presented in a transparent manner, supported by evidence from the text. Furthermore, the model’s reliance solely on textual data means it may not capture DDI information available in other modalities, such as molecular structures or patient-specific genomic data. Addressing these limitations in future work is crucial for fully realizing the framework’s value in personalized and precision medicine.

Ultimately, this framework can serve as a foundational component in next-generation biomedical text mining pipelines, complementing structured databases and enhancing the situational awareness of clinicians and drug safety professionals alike.

## Conclusion

This paper introduced BioMCL-DDI, a unified meta-contrastive learning framework for few-shot drug–drug interaction (DDI) extraction. It performs a fine-grained, multi-class classification of drug pairs into five distinct DDI types: DDI-false, DDI-effect, DDI-mechanism, DDI-advise, and DDI-int. By jointly optimizing prototypical classification and instance-level contrastive learning within a fully supervised setting, BioMCL-DDI achieves robust performance under data-scarce conditions without relying on episodic task construction or pretraining. Our extensive evaluations on DDI-2013 and DDI-DrugBank benchmarks demonstrate consistent improvements over state-of-the-art baselines, with strong generalization across domains and DDI subtypes. More importantly, evaluation on the recent TAC 2018 DDI Extraction dataset confirms that BioMCL-DDI maintains state-of-the-art performance under substantial domain shift. This robustness highlights the model’s applicability to real-world biomedical texts such as structured product labels and regulatory documents, which are central to pharmacovigilance practice. The proposed framework not only enhances class-level alignment and inter-class discrimination but also offers training scalability and architectural simplicity—key traits for integration into real-world pharmacovigilance pipelines. Moreover, BioMCL-DDI remains effective despite severe class imbalance, highlighting its applicability to rare but clinically significant interactions.

In future work, we aim to improve the interpretability and adaptability of BioMCL-DDI by integrating external biomedical knowledge, exploring zero-shot or continual learning settings, and extending support for multi-label or nested DDI relations. Another promising direction is to incorporate advanced graph neural networks (GNNs) for DDI prediction. Recent studies have demonstrated the effectiveness of GNNs in modeling complex biomedical relationships such as molecular interaction networks and drug–target associations [60,61]. By combining BioMCL-DDI’s sentence-level contextual embeddings with graph-based relational representations, future extensions could capture higher-order dependencies among drugs, targets, and interactions, thereby improving robustness and generalizability across heterogeneous biomedical corpora. Ultimately, we envision this framework contributing to the development of scalable, data-efficient, and clinically deployable decision support tools for personalized drug safety assessment.
